# Macromolecular Viral Entry Inhibitors as Broad‐Spectrum First‐Line Antivirals with Activity against SARS‐CoV‐2

**DOI:** 10.1002/advs.202201378

**Published:** 2022-05-11

**Authors:** Rüdiger Groß, Lívia Mesquita Dias Loiola, Leila Issmail, Nadja Uhlig, Valentina Eberlein, Carina Conzelmann, Lia‐Raluca Olari, Lena Rauch, Jan Lawrenz, Tatjana Weil, Janis A. Müller, Mateus Borba Cardoso, Andrea Gilg, Olivia Larsson, Urban Höglund, Sandra Axberg Pålsson, Anna Selch Tvilum, Kaja Borup Løvschall, Maria M. Kristensen, Anna‐Lena Spetz, Fortune Hontonnou, Marie Galloux, Thomas Grunwald, Alexander N. Zelikin, Jan Münch

**Affiliations:** ^1^ Institute of Molecular Virology Ulm University Medical Center Ulm 89081 Germany; ^2^ Department of Chemistry and iNano Interdisciplinary Nanoscience Centre Aarhus University Aarhus 8000 Denmark; ^3^ Brazilian Synchrotron Light Laboratory Brazilian Center for Research in Energy and Materials Campinas São Paulo 13083‐970 Brazil; ^4^ Fraunhofer Institute for Cell Therapy and Immunology IZI Leipzig 04103 Germany; ^5^ Adlego Biomedical AB Solna 171 65 Sweden; ^6^ Department of Molecular Biosciences The Wenner‐Gren Institute Stockholm University Stockholm 10691 Sweden; ^7^ Université Paris‐Saclay INRAE, UVSQ, VIM Jouy‐en‐Josas 78352 France

**Keywords:** broad‐spectrum antivirals, entry inhibitors, in vivo, macromolecules, polyanions, respiratory syncytial virus (RSV), SARS‐CoV‐2

## Abstract

Inhibitors of viral cell entry based on poly(styrene sulfonate) and its core–shell nanoformulations based on gold nanoparticles are investigated against a panel of viruses, including clinical isolates of SARS‐CoV‐2. Macromolecular inhibitors are shown to exhibit the highly sought‐after broad‐spectrum antiviral activity, which covers most analyzed enveloped viruses and all of the variants of concern for SARS‐CoV‐2 tested. The inhibitory activity is quantified in vitro in appropriate cell culture models and for respiratory viral pathogens (respiratory syncytial virus and SARS‐CoV‐2) in mice. Results of this study comprise a significant step along the translational path of macromolecular inhibitors of virus cell entry, specifically against enveloped respiratory viruses.

## Graphical abstract

Macromolecules are shown herein to act as broadly acting antiviral agents, most importantly against severe acute respiratory syndrome coronavirus 2 (SARS‐CoV‐2), by interacting with the viruses thereby inhibiting viral entry. Antiviral activity is demonstrated in vitro against a panel of viruses, and in vivo against two respiratory pathogens (respiratory syncytial virus (RSV) and SARS‐CoV‐2). With due optimization, polymers hold translational promise as first‐line broad‐spectrum antivirals.

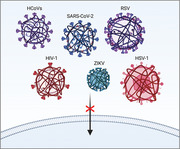

## 1 Introduction

The past few decades have witnessed several pandemics caused by viral pathogens. Of these, the ongoing SARS‐CoV‐2 pandemic is one of the deadliest and the one associated with the highest socioeconomic burden. It has provided an incredible boost to the development and testing of vaccines, but at the same time underscored the current limitations in the field of antiviral drugs. Developments of antiviral compounds, from virtually barren field to over a hundred marketed compounds in just over 40 years,^[^ ^1^ ^]^ is a story of an incredible success of medicinal chemistry. Nevertheless, recurring pandemics highlight the need to develop broad‐spectrum antiviral agents.^[^ ^2^, ^3^, ^4^ ^]^ The so‐called “one bug—one drug” approach that dominates antiviral therapies today is limited and offers little to the preparedness of the society to (re)emerging pathogens.

A class of antiviral agents with a unique broad spectrum of antiviral activity is based on polymers. Macromolecular antiviral agents have been known almost as long as polymers as chemical entities.^[^ ^5^, ^6^, ^7^ ^]^ Much like neutralizing antibodies, polymers can interact with the viral surface and in doing so prevent virus interaction with the cell. Antiviral polymers can be positively or negatively charged, of biological or synthetic origin. The addition of hydrophobic groups to the polymer structure offers a unique opportunity to create virucidal agents that impart permanent damage to the virion.^[^ ^8^, ^9^ ^]^ For an exhaustive, inclusive coverage of the subject we refer to a suite of excellent reviews that appeared in the literature in recent months.^[^ ^10^, ^11^, ^12^, ^13^, ^14^, ^15^ ^]^

Specifically, with regards to SARS‐CoV‐2, the past several months documented important evidence of the activity of polymeric antivirals on this virus, which include an observation of rapid virus inactivation by an anionic polymer, toward the design of disinfecting surfaces.^[^ ^16^ ^]^ SARS‐CoV‐2 was also effectively inhibited by the cationic chitosan derivative^[^ ^17^ ^]^ and conjugated polymers,^[^ ^18^ ^]^ cationic or anionic, which exhibited light‐induced antiviral activity. Polysulfates, specifically those based on linear or hyperbranched polyglycerols, blocked SARS‐CoV‐2 infectivity via electrostatic interactions and were superior to heparin in terms of active concentration (and also exhibit lower anticoagulant activity).^[^ ^19^ ^]^ Similarly, sulfonate‐decorated gold nanoparticles and cyclodextrins were also effective as anti‐SARS agents.^[^ ^20^ ^]^ In our recent work, we showed that sulfonated carrageenans are active as inhibitors of SARS‐CoV‐2 infectivity and identified commercial sprays already found on the market and are thus readily available as the first‐line defense against the current viral pandemic.^[^ ^21^ ^]^ Furthermore, we showed that heparin, a natural sulfonated polymer, has a unique multiarm activity that encompasses direct antiviral effects as well as the highly beneficial anticoagulant and anti‐inflammatory effects.^[^ ^22^ ^]^

An essential characteristic of polymeric, and especially polyanionic antivirals is that these agents often exhibit a broad‐spectrum antiviral activity. This notion was revealed decades ago, and since then has been documented for a highly diverse range of polymers and nanoparticles.^[^ ^7^, ^14^, ^23^ ^]^ The interaction of polymers with the virion surface is due to the electrostatic attraction between the polymer and the amino acids in the viral proteins, and possibly involves hydrophobic interactions with the protein's inner volume and/or the lipid bilayer comprising the viral envelope.^[^ ^7^ ^]^ Polymeric antivirals are macromolecules consisting of a high number of repeat units and can optimize interaction with the viral surface through conformational changes, adapting it to virtually any virus.

In previous work, we analyzed a panel of polymers against a range of viral pathogens, and this allowed us to find structure–activity relationships and address two equally important questions: i) which polymer(s) have a broad spectrum of antiviral activity, and ii) which viruses are most susceptible to inactivation by polymeric antivirals.^[^ ^24^ ^]^ Lead polymers identified in our studies were united by structure in being sulfonates or carboxylates with well‐defined hydrophobicity of the macromolecular backbone. Viral pathogens were easiest to counteract if the envelope was exposed (sparse glycoprotein coverage, e.g., HIV‐1) but less susceptible to inhibition by polymers if the glycoprotein coverage was dense (e.g., the Zika virus).^[^ ^24^ ^]^

An outstanding question with regard to the translational path of the polymeric antivirals relates to their mode of use. Systemic application of synthetic polyanions as a treatment against HIV‐1 has failed in clinical trials due to a lack of efficacy and strong safety concerns.^[^ ^25^ ^]^ In contrast, a formulation of dendritic polyanions is successful as a topical microbicide, specifically for concurrent activity against multiple sexually transmitted viruses.^[^ ^26^ ^]^ The overarching goal of this study is to develop broad‐spectrum antiviral agents against viral infections affecting the respiratory tract and specifically against SARS‐CoV‐2, to be administered via nasal/oral spray or nebulizers, for a localized activity against respiratory pathogens. This endeavor is not without precedent and e.g. nasal/throat sprays containing negatively charged carrageenans are available commercially as antivirals, although clinical trials showed modest if any direct antiviral effects by these agents.^[^ ^27^ ^]^ Macromolecular antivirals identified in our prior in vitro studies,^[^ ^24^ ^]^ specifically polystyrene sulfonate (PSS), were potent and safe. Here, we analyze their potential for application against respiratory viral pathogens in vivo.

Toward the overall goal, we synthesized PSS with distinctly different molar masses, additionally formulated these polymers as a shell around spherical gold nanoparticles of several sizes (5 to 40 nm) to enhance the multivalency effects, quantified antiviral effects of the antivirals on several viruses in vitro, performed a detailed study into the mode of interaction of the leads with SARS‐CoV‐2 and lentiviral particles. Furthermore, we performed an in‐depth toxicity evaluation of the antivirals upon nasal instillation in mice and quantified antiviral effects in vivo against RSV and SARS‐CoV‐2 in mice. Taken together, the results of this study comprise a significant step along the translational path of macromolecular inhibitors of virus cell entry, specifically against respiratory viruses.

## 2 Results and Discussion

**Figure 1:**
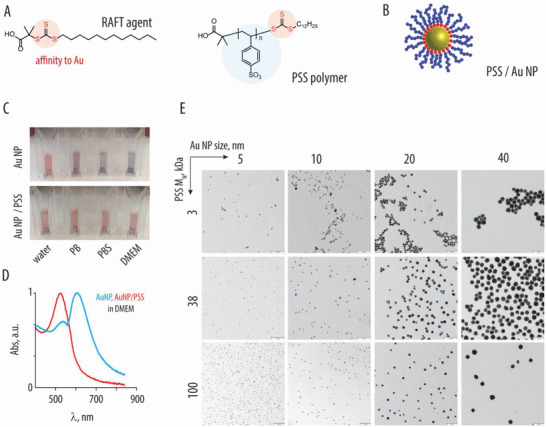
A) Chemical formula of the RAFT agent and the resulting polystyrene sulfonate polymer (PSS). B) Schematic illustration depicting association of the RAFT‐derived polymers (blue) with gold nanoparticles, due to affinity of the trithiocarbonate (red) to the gold surfaces. C) Photography images of the 10 nm gold nanoparticles, with or without the brush‐type corona comprised of PSS (100 kDa) in water, phosphate buffer, phosphate buffer saline, or DMEM cell culture medium: colloidally dispersed AuNP have a characteristic red color, whereas nanoparticle aggregation produces a characteristic blue color. D) UV/vis spectra corresponding to the images in panel C for AuNP with or without PSS corona in DMEM. E) Transmission electron microscopy image for AuNP (sized 5–40 nm) functionalized with PSS (3, 38, and 100 kDa), scale bar = 100 nm.

Macromolecular inhibitors based on PSS were synthesized via the reversible addition–fragmentation chain transfer (RAFT) polymerization (**Figure** **1**).^[^ ^28^ ^]^ This polymer was chosen based on our previous report on the antiviral activity of polyanions.^[^ ^24^ ^]^ Monomer‐to‐RAFT agent ratio was used as a tool to obtain polymers of molar masses of 3, 38, and 100 kDa.

Recent evidence illustrates that the 3D architecture of macromolecular or supramolecular antiviral agents is an important factor in its activity.^[^ ^23^, ^29^, ^30^ ^]^ Specifically, the nanoparticle size has been shown to play a significant role in its virus inhibition activity.^[^ ^31^ ^]^ RAFT polymerization is based on the chain transfer reagents containing sulfur (dithiocarbonates, trithiocarbonates)^[^ ^28^ ^]^ and produces macromolecules that are readily useful for modifying gold nanoparticles.^[^ ^32^, ^33^, ^34^ ^]^ The latter have a wide range of applications in biotechnology and biomedicine,^[^ ^35^, ^36^ ^]^ including the design of antiviral agents.^[^ ^23^ ^]^ To broaden the scope of this study, we immobilized PSS onto spherical gold nanoparticles sized from 5 to 40 nm (Figure 1B). For brevity, nanoparticle/ polymer formulations are denoted using a 3‐digit code whereby the first letter corresponds to the gold nanoparticle size (A‐D), second digit *S* denotes PSS (S), and the third digit (1‐3) indicates the polymer molar mass, e.g., 5 nm particles decorated with the 3 kDa sample of PSS is denoted AS1. Core–shell nanoparticles were prepared by coincubating Au nanoparticles (AuNP) with excess polymer followed by purification via spin filtration. Filtrate solutions were monitored via UV–vis spectrometry to confirm the completeness of removal of the nonbound, excess polymer. The resulting core–shell nanoparticles were evaluated in terms of colloidal stability, which is a critical asset for their biomedical applications (Figure 1C–E). Particle functionalization with PSS afforded nanomaterials with excellent colloidal stability, illustrated by UV–vis spectra (Figure 1D) and the transmission electron microscopy images (Figure 1E). These particles could be freeze‐dried and reconstituted to well‐dispersed formulations in water, phosphate‐buffered saline, and/or cell culture medium supplemented with serum (Figure 1C,D).

**Figure 2:**
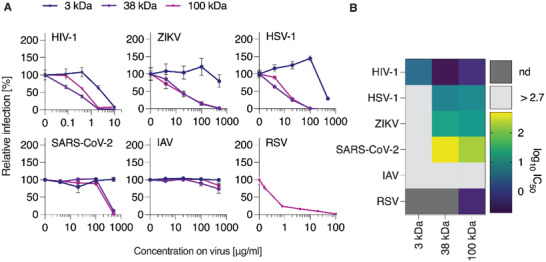
Broad‐spectrum antiviral activity of PSS polymers with variable length. A) PSS compounds with molar masses of 3, 38, and 100 kDa were dissolved and titrated in PBS before adding virus at 1:1 (v/v) dilution, resulting in the indicated concentrations. Following incubation at 37 °C for 30 min, mixtures were added to target cells (HIV‐1: TZM‐bl, ZIKV: VeroE6, HSV‐1: ELVIS, SARS‐CoV‐2: Caco‐2, RSV: A549, IAV: MDCK). Infection rates were determined one (HSV‐1, RSV) or two days postinfection (all other viruses) by quantification of reporter cell line activity (HIV‐1, HSV‐1), immunodetection of viral proteins (ZIKV, SARS‐CoV‐2), flow‐cytometric analysis of virally expressed reporter GFP (RSV) or neuraminidase activity assay (IAV). Raw values were baseline‐subtracted (uninfected cells) and normalized to cells infected without polymer pre‐treatment of virus. Shown are mean values obtained from two (*n* =2, for HIV‐1, ZIKV, SARS‐CoV‐2) or three (*n* = 3, for HSV‐1, IAV) independent experiments each performed in triplicates ± SEM. Data for RSV are shown as mean values from three independent experiments (*n* = 3) in duplicates ± SEM. B) IC_50_ values for each virus and polymer are shown as log_10_ transform to highlight differences, calculated from nonlinear regression curve fits (inhibitor vs. normalized response, GraphPad Prism).

To assess the potential of the synthesized PSS polymers as antiviral agents, we first quantified their activity as inhibitors of viral infection in cell culture, using a panel of enveloped viruses. Viruses were preincubated with the polymers and used to infect susceptible target cells. Infection rates were determined one to two days post‐infection. Data shown in **Figure** **2** confirmed the previous findings from us^[^ ^24^ ^]^ and others^[^ ^37^ ^]^ and demonstrate that PSS inhibits infectivity of a broad panel of enveloped viral pathogens, which includes the human immunodeficiency virus (HIV‐1), Zika virus (ZIKV), herpes simplex virus 1 (HSV‐1), and respiratory syncytial virus (RSV). The range of viruses inhibited by PSS also included SARS‐CoV‐2, which is of the highest value under the circumstances of the current pandemic. Although inhibition in this case was significantly less potent than that in the case of HIV‐1, the potency‐related IC_50_ value was nevertheless under 1 g L^−1^ (Figure 2A). No cytotoxicity was observed at doses required to reach full inhibition and only moderate effects on cellular metabolic activity were observed with maximally possible doses of up to 1.75 mg mL^−1^ on cells (Figure S1, Supporting Information). One viral pathogen that was not inhibited by PSS was the influenza A virus (IAV). This could be due to the fact that IAV has an affinity to sialic acid but not polyanionic heparan sulfate,^[^ ^7^ ^]^ which is commonly used by most enveloped viruses for cellular interaction. From the standpoint of the structure–activity relationship, a strong conclusion from the IC_50_ values shown in Figure 2B is that the polymer chains with the lowest molar mass (3 kDa) are hardly active as antiviral agents. In contrast, average molar mass 38 and 100 kDa polymer samples were effective and rather similar in their antiviral activity.

**Figure 3:**
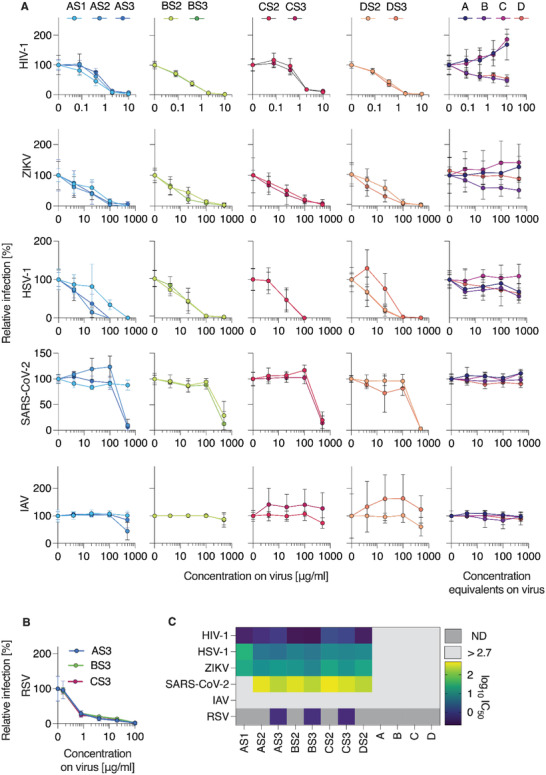
Broad‐spectrum antiviral activity of AuNP‐coupled PSS. A) Compounds were dissolved and titrated in PBS before adding virus at 1:1 dilution, resulting in the indicated concentrations on virus. Following incubation at 37 °C for 30 min, mixtures were added to target cells at tenfold dilution (HIV‐1: TZM‐bl, ZIKV: VeroE6, HSV‐1: ELVIS, SARS‐CoV‐2: Caco‐2, RSV: A549, IAV: MDCK). Infection rates were determined one (HSV‐1, RSV) or two days after infection (all others) by measuring enzyme activity of the reporter cell line (HIV‐1, HSV‐1), immunodetection of viral proteins (ZIKV, SARS‐CoV‐2) or neuraminidase activity assay (IAV). Raw values were baseline‐subtracted (uninfected cells) and normalized to cells infected without polymer pre‐treatment of virus. Shown are mean values obtained from two (*n* = 2, for HIV‐1, ZIKV, SARS‐CoV‐2) or three (*n* = 3, for HSV‐1, IAV) independent experiments each performed in triplicates ± SEM. B) Activity of S3‐based AuNP‐coupled polymers against RSV measured by flow‐cytometric analysis of virally expressed reporter GFP. Data are shown as mean values from three independent experiments (*n* = 3) in duplicates ± SEM. C) IC_50_ values for each virus and polymer are shown as log_10_ transform to highlight differences, calculated from nonlinear regression curve fits (inhibitor vs normalized response, GraphPad Prism).

Next, we determined the antiviral activity of the gold nanoparticles decorated with a PSS brush‐type corona (**Figure** **3**). Antiviral activity of the polymer was successfully endowed to the AuNP and formulations were efficacious inhibitors of infectivity for HIV‐1, HSV‐1, ZIKV, RSV, and SARS‐CoV‐2, with the overall activity profile being rather similar to the polymer itself (Figure 3A,B). Specifically, inhibition of HIV‐1 was the most potent, whereas inhibition of SARS‐CoV‐2 was significantly less potent (Figure 3A,C). With regard to the AuNP size, we observed rather little variation in the antiviral activity, which suggests that the polymeric brush‐type corona negates the effects of the particle curvature on the binding of the viral pathogens. An important observation is that immobilization onto AuNP achieved a significant potentiation of the antiviral effects for the lowest molar mass PSS. Specifically, 3kDa PSS as a polymer was ineffective as an antiviral (Figure 2), yet formulations of this polymer on AuNP proved to be effective inhibitors of infectivity of HIV‐1, HSV‐1, and even ZIKV (the latter has previously proven to be amongst the viruses most resilient to the polymer‐based infectivity inhibition.^[^ ^24^ ^]^) For HIV‐1, potentiation due to immobilization onto AuNP was as high as 10‐fold (from 3 mg L^−1^ for the polymer to 0.3 mg L^−1^ for the polymer on the 5 nm nanoparticles, Figure 3A,C). None of the AuNP‐conjugated polymers exerted cytotoxic activity at doses tested for antiviral activity (Figure S1, Supporting Information). It is worthy of note that unlike prior reports,^[^ ^31^ ^]^ our data reveal minor if any difference in the antiviral activity for the core–shell nanoparticles depending on the size of the core AuNP. A plausible explanation for this is that instead of a defined contact surface,^[^ ^31^ ^]^ the core–shell particles used in this work present an adaptable brush with multiple polymer chains, each consisting of multiple sulfonated monomer units. We believe that this is why the nominal nanoparticle size has not revealed itself as a decisive factor in the virus inhibition studies. Taken together, the results presented above illustrate that PSS and its formulations on AuNP are broad‐spectrum antiviral agents, acting on a range of enveloped viruses including SARS‐CoV‐2. Next steps in our studies were focused on the in‐depth characterization of antiviral effects against this virus, in vitro and in vivo.

**Figure 4:**
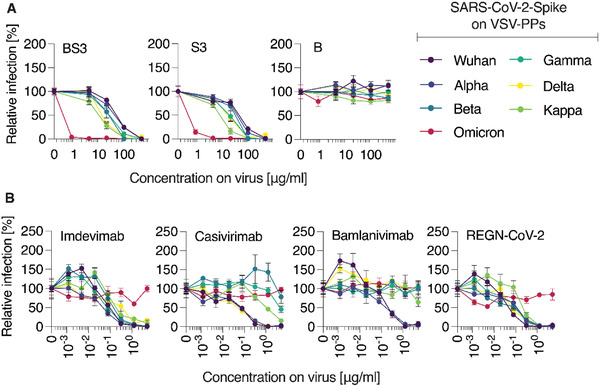
Inhibition of SARS‐CoV‐2 Spike pseudoparticles by A) PSS and AuNP‐PSS and B) specific mAbs. Compounds or mAbs were serially titrated in PBS before adding pseudoviruses containing Spike of indicated SARS‐CoV‐2 VOC 1:1, resulting in indicated concentrations on virus. After 30 min at 37 °C, mixtures were added to Vero E6 cells. Pseudoparticle entry was evaluated by Firefly luciferase measurement 16–18 h post‐transduction. Values were background‐subtracted (uninfected cells) and normalized to cells infected in absence of inhibitors. Shown are mean values obtained from three independent experiments (*n* = 3) each performed in triplicates ± SEM for (A), one experiment in triplicates (*n* = 1) for mAbs tested as conformation of VOC immune‐evading behavior in (B).

One of the highest points of concern during the current pandemic is the continued evolution of the virus and the emergence of multiple SARS‐CoV‐2 variants of concern (VOC). Broad‐spectrum activity of the macromolecular inhibitors of virus cell entry suggests that these agents should be active against the diverse variants. To validate this, rhabdoviral pseudoparticles were engineered to contain the SARS‐CoV‐2 spike proteins corresponding to a range of VOC (Wuhan, Alpha, Beta, Gamma, Delta, Kappa, Omicron). Monoclonal antibodies (Imdevimab, Casivirimab, Bamlanivimab) and the combination of antibodies known as REGN‐CoV‐2 were used as control inhibitors of virus cell entry and VOC‐specific immune evasion. Biological inhibitors are being developed as highly specific therapeutics and only with special engineering can these be broadly neutralizing. Indeed, in our hands, two antibodies showed limited spectrum of activity against VOC, while the antibody cocktail as well as Imdevimab were active against all the VOC tested in this work except for Omicron, confirming previous data (**Figure** **4** **)**.^[^ ^38^, ^39^, ^40^, ^41^ ^]^ The macromolecular inhibitors S3 (PSS 100 kDa polymer solution) and BS3 (100 kDa PSS polymer immobilized on the 10 nm AuNP) were active against all VOC. These data illustrate that once developed, the antiviral formulations based on PSS are very likely to be active against future emerging VOC.

**Figure 5:**
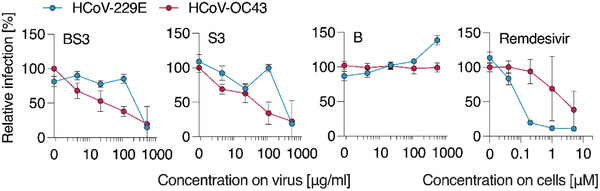
Inhibition of common cold coronavirus HCoV‐229E and‐OC43 by PSS and AuNP‐PSS. Compounds were titrated in PBS and mixed with virus at 1:1 dilution (v/v), resulting in indicated concentrations (for PSS). After incubation at 37 °C for 30 min, mixtures were added to Huh7 cells. After two (229E) or three (OC43) days, infection was quantified by immunodetection of viral N protein. Values of infected cells were subtracted and infection rates normalized to cells infected in the absence of compounds. Data from three independent experiments (*n* = 3) in triplicates, means ± SEM.

The spectrum of activity of the macromolecular inhibitors also covers endemic seasonal human coronaviruses HCoV‐229E and OC43 (**Figure** **5**). For both viral pathogens, the polymer and the core–shell nanoformulation were effective as inhibitors of viral entry. The relative efficacy of inhibition between HCoV‐229E and ‐OC43 for the macromolecular inhibitors appears to be the opposite of that for remdesivir, the latter being significantly less efficacious against the HCoV‐OC43 virus whereas PSS is less efficacious against the HCoV‐229E. Nevertheless, most importantly, at the highest inhibitor concentration used, we observed strong inhibition of viral infectivity for both pathogens, at concentrations similar to those required for inhibition of SARS‐CoV‐2 (Figures 2 and 3).

**Figure 6:**
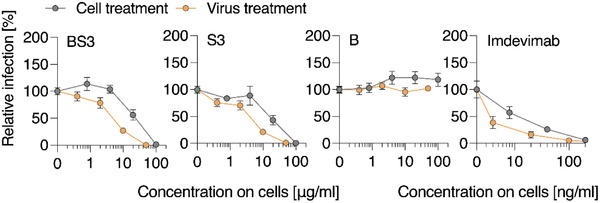
Effect of cell treatment versus virus treatment with polymers on SARS‐CoV‐2‐Spike driven entry. Compounds were titrated and added to VeroE6 cells, which were then incubated for 60 min at 37 °C and subsequently infected with Wuhan Spike‐VSV‐pseudoparticles (“Cell treatment”‐grey circles). Alternatively, in the “Virus treatment” (yellow circles) conditions, virions and compounds were premixed, incubated for 30 min at 37 °C and then the mixture was added to cells at tenfold dilution. Shown are mean values obtained from three independent experiments (*n* = 3) each performed in triplicates ± SEM.

To investigate the mechanism of antiviral activity, virus inhibition studies were carried out via the “virus treatment” or “cell treatment” protocols, which refers to the pre‐incubation of the polymer with the virus or the cells, respectively. These experiments illustrate that pretreatment of the virus affords a lower active antiviral concentration (**Figure** **6**), as is expected for the inhibitors of virus cell entry that need to interact with the viral particles to exert their effect.

**Figure 7:**
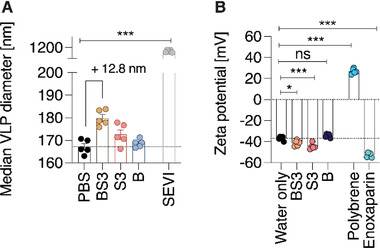
Nanoparticle tracking analysis reveals biophysical changes in virus‐like particles following incubation with AuNP‐PSS and free PSS. Fluorescent lentiviral VLPs were used to observe the binding of AuNP‐PSS and PPS to viral particles by fluorescent nanoparticle tracking analysis. After incubating VLPs with 100 µg mL^−1^ BS3, S3 or B for 60 min at 37 °C, A) the median VLP diameter was determined. PBS was used as a negative control, while peptide nanofibrils aggregating virions (SEVI, 100 µg mL^−1^) were used as a positive control. B) Additionally, pre‐incubated VLPs were diluted in water to determine the surface charge by measuring zeta potential. Polycation polybrene and polyanion enoxaparin were used as controls to induce strong changes in virion surface charge in a positive and a negative direction, respectively. Both parameters were acquired by F‐NTA at 488 nm using a ZetaView TWIN, thus excluding scattering signals from polymers, AuNP or non‐VLP particles contained in the medium. Five acquisitions (*n* = 5) were performed for each compound, values show mean values ± SEM. One‐way‐ANOVA with Dunnett's post‐test compares median VLP diameter and Zeta potential to PBS/water treated VLPs. * = *p* < 0.05, *** = *p* < 0.001, all without indication non‐significantly different from PBS/water only.

As further proof of the direct polymer–virion interaction, nanoparticle tracking analysis of polymer‐treated virus‐like particles was conducted. Lentiviral particles were used due to the availability of fluorophore‐conjugated virus‐like‐particles (MLVgagYFP‐VLPs),^[^ ^42^ ^]^ which are trackable in fluorescence NTA (F‐NTA) thus excluding scattering signals from polymers or AuNPs themselves. As Gag‐based VLPs, these particles do not contain any viral glycoproteins on their surface and interactions with polymers are thus either mediated by membrane lipids or host cell proteins incorporated into the membrane. Lentiviral particles incubated with BS3, but not S3 or the B only, showed a notably higher median diameter (ca. 12 nm), suggesting direct binding of the AuNP–PSS to virions. However, PSS did not lead to virion aggregation as observed for the control substance SEVI (peptide nanofibrils, aggregating virions as µm‐sized clusters, **Figure** **7**A).^[^ ^43^ ^]^ In agreement with virion‐binding, the surface charge (zeta potential) of the same VLPs significantly decreased to more negative values (−41 mV for BS3, −45 mV for S3) from baseline (−37 mV) after incubation with S3 and BS3, but not B only (Figure 7B).

**Figure 8:**
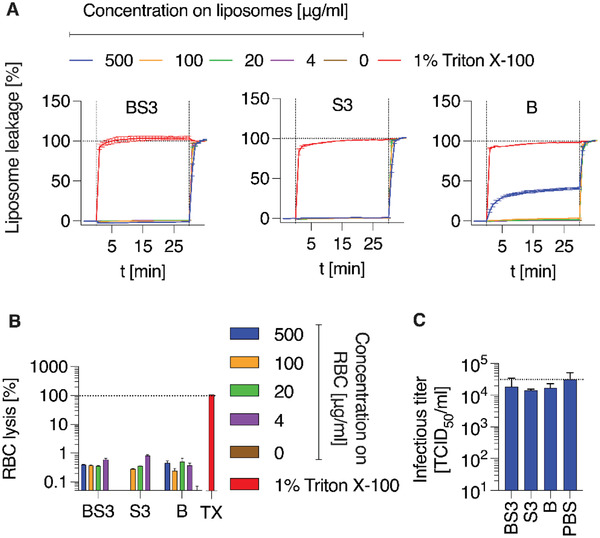
Evaluation of membrane‐lytic/virucidal activity of AuNP‐PSS and PSS. (A) Membrane‐disrupting activity of BS3, S3 and B evaluated by liposome leakage assay. Virus‐like‐liposomes (DOPC/SM/Chol; 45/25/30 mol%) filled with carboxyfluorescein (50 × 10^−3^ m) and purified by SEC, were added to 96‐well black side‐clear bottom plates and baseline fluorescence recorded for 5 min once per minute. Compounds were then added at indicated concentration (first vertical line) and fluorescence increase (indicating membrane disruption) recorded for 30 min once per minute. Triton X‐100 was then added to all wells at 1% final concentration to induce total lysis (second vertical line). Percent values shown are background‐subtracted (signal of liposome prior to addition of compounds) and normalized to fluorescent signal achieved in each well after complete liposome leakage induced by addition of 1% (v/v) Triton X‐100. Magnitude of relative leakage prior to addition of Triton X‐100 thus reveals the membrane‐disrupting activity of the tested compounds. Two independent experiments (*n* = 2) in triplicates, means ± SEM. B) Membrane‐disrupting activity by BS3, S3 and B evaluated by red blood cell lysis assay. 3×10^6^ RBC were incubated with indicated concentrations or 1% Triton X‐100 (colors as in A) for 30 min, samples then centrifuged for 5 min at 500 × *g*, supernatants transferred to new plates and released hemoglobin measured via absorbance at 450 nm. Blank values (PBS only) subtracted, normalized to values after addition of 1% Triton X‐100. One experiment (*n* = 1) in triplicates, means ± SEM. C) SARS‐CoV‐2 virus stocks were incubated with 500 µg mL^−1^ of compounds for 30 min at 37 °C before residual infectivity was determined by TCID_50_ endpoint titration according to Reed & Muench. Two independent experiments (*n* = 2) in triplicates, means ± SEM.

We then investigated the potential membranolytic behavior of PSS, AuNP and their core–shell formulation with model, virus‐like liposomes and red blood cells (RBC) and observed that both liposomal and RBC membranes were not compromised by these agents (**Figure** **8**A,B). This suggests that macromolecular inhibitors of virus cell entry used in this work are not virucidal, but are virustatic by the mechanism of activity. This conclusion is directly confirmed by the quantifying viral titers of SARS‐CoV‐2 after treatment with PSS and AuNP‐PSS, which proved to be largely unchanged upon the interaction with PSS (Figure 8B). We envision that virucidal behavior, which could be beneficial for an antiviral formulation,^[^ ^23^ ^]^ can be engineered into PSS with the use of RAFT agents that contain hydrophobic (e.g., cholesterol) groups.^[^ ^9^ ^]^ However, lack of virucidal, membranolytic activity possibly comes with an advantage of reduced cellular toxicity, rendering these compounds very promising leads for further antiviral drug development.

The above experiments present PSS and its core–shell formulation based on AuNP as broad‐spectrum antiviral agents including activity against SARS‐CoV‐2. Successful translation from lab to clinic requires a rigorous evaluation of antiviral agents in vivo, starting with the toxicity evaluation. The overarching goal of this study is to develop the formulation of PSS, as a soluble polymer or anchored onto AuNP, as a nasal/oral spray for localized antiviral therapy or prophylaxis. An attractive feature of such formulations is that potential side effects can be expected to be only localized, rather than systemic, as typically seen for the nonabsorbing drugs.^[^ ^44^ ^]^

**Figure 9:**
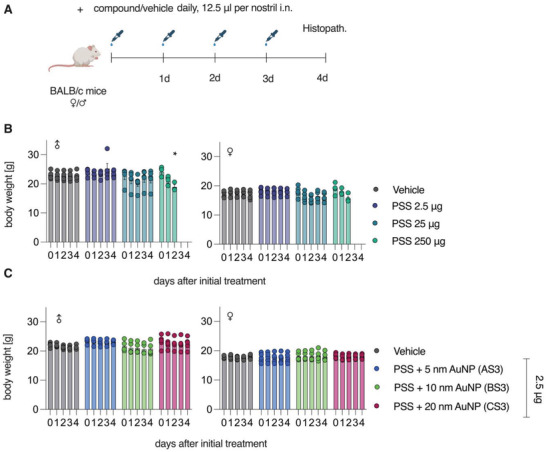
In vivo tolerability study of PSS and AuNP‐PSS in BALB/c mice. A) Experimental setup for tolerability study. BALB/c mice (5 male and 5 female/group; *n* = 5) received indicated amounts of compound once daily by intranasal application, with the total dose split to both nostrils. B,C) Animals were weighed daily. At 24 h after the final application, animals were sacrificed and nasal cavity, olfactory bulb, trachea, lung, esophagus, and stomach were analyzed by histology. Data presented as means ± SEM, *n* = 5 (lower for timepoints where some animals were already sacrificed). Significance in weight differences between vehicle and PSS‐treated mice for each time point tested using ordinary one‐way ANOVA with Dunnett's post‐test, * = *p* < 0.05. All without indication are not significantly different from vehicle. Schematic in (A) created with BioRender.com.

**Figure 10:**
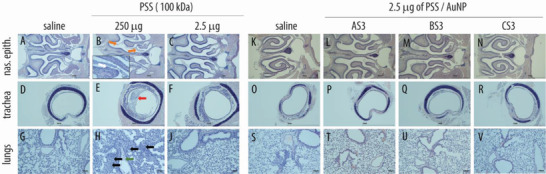
Histopathological evaluation in respiratory organs following administration of PSS (A–J) and core–shell PSS‐AuNP nanoformulations (K–V). Images depict nasal epithelium (A–C; K–N), trachea (D–F; O–R), and lung tissue (G–J; S–V) for vehicle controls (saline, panels A, D, G, K, O, S), 100 kDa PSS dosed at 250 µg (highest feasible dose, HFD, panels B, E, H) or 2.5 µg (highest tolerable dose, panels C, F, J); AS3 (L, P, T); BS3 (M, Q, U) and CS3 (N, R, V) dosed at 2.5 µg. For PSS and core–shell nanoformulations dosed at 2.5 µg, no histopathological changes, as compared to vehicle, were evident.

As the first toxicity evaluation, we performed a tolerability study using solutions of 100 kDa PSS (**Figures** **9** and **10**). Polymer solutions were dosed intranasally into mice once daily for 4 d, after which the animals were sacrificed (Figure 9A).

Higher doses (250 µg, highest feasible dose, HFD) were associated with a substantial decline in body weight, as well as decreased health status, as evidenced by presentation of piloerection and hunched posture, and all animals in the 250 µg group were euthanized on day 3 due to severe loss in body weight (Figure 9B). Histopathological analysis revealed with moderate granulocytic inflammation in the lungs and nasal cavity in these dose groups (Figure 10).

Specifically, intraluminal fluid and debris (Figure 10B orange arrows) and erosions of the olfactory epithelia and serofibrinous exudate (Figure 10B, white arrow in the panel inset) were evident in the nasal passage of the HFD group. In addition, intraluminal mucous with inflammatory cells was evident in the trachea of the HFD group (Figure 10E, red arrow). Finally, alveolar fluid (Figure 10H, green arrow) and alveolar scattered granulocytes (Figure 10H, black arrows) were evident in the lungs of the HFD group. In contrast, PSS dose of 2.5 µg (12.5 µL of 0.1 g L^−1^ solution) was associated with no animal body weight loss (Figure 9B); histopathology observations of the harvested tissues revealed no signs of the localized toxicity at this administered dose (Figure 10C,F,J). At a dose of 2.5 µg, core–shell formulations based on PSS (100 kDa) and nanoparticle sized between 5 and 20 nm were also tolerated without signs of toxicity, as judged by the body weight measurements (Figure 9C) and the histopathology observations (Figure 10K–V).

**Figure 11:**
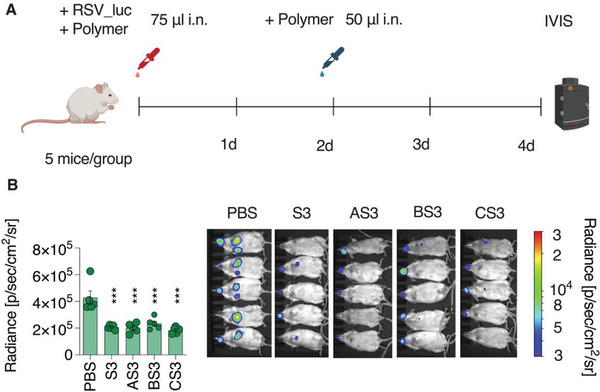
In vivo inhibition of RSV infection by PSS and its core–shell formulations with AuNP sized 5, 10, and 20 nm. A) Female BALB/c mice (*n* = 5 per group) were infected by intranasal administration of rHRSV‐Luc (10^5^ pfu) pre‐incubated with 0.1 g L^−1^ polymers. Mice were treated at two dpi before visualization and B) quantification by the luminescence of rHRSV‐Luc replication in mice at four dpi. Values are shown as means ± SEM. Ordinary one‐way ANOVA with Dunnett's post‐test compares mean radiance of treated animal groups to PBS‐treated control animals. *** = *p* < 0.001. pfu, plaque‐forming units; dpi, days post‐infection. Schematic in (A) created with BioRender.com.

In vivo evaluation of the antiviral effects was conducted in mice using RSV and SARS‐CoV‐2 as pathogens. In the case of RSV, a recombinant virus expressing the luciferase (rHRSV‐Luc)^[^ ^45^ ^]^ was incubated with the inhibitors (0.1 g L^−1^) for 30 min before intranasal administration at a dose equivalent to 2.5 µg polymer (**Figure** **11**). At two days postinfection (dpi), a second dose of polymer (2.5 µg) was administrated (Figure 11A). Viral replication in mice was quantified at four dpi by luminescence measurement using IVIS imaging system (Figure 11B). At four dpi, each of the four antiviral formulations exhibited statistically significant inhibition of virus replication (Figure 11B).

**Figure 12:**
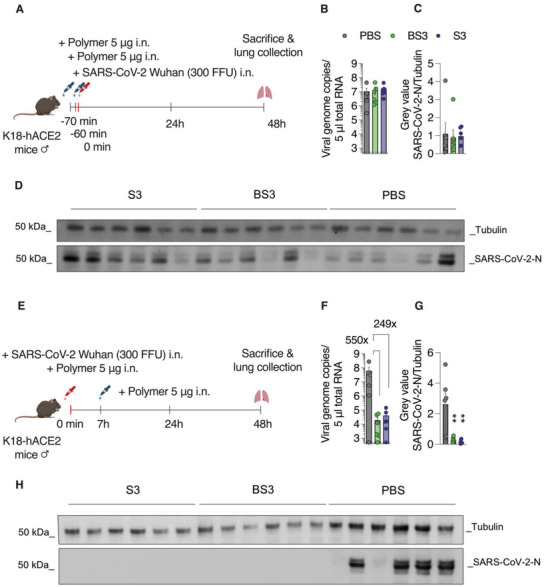
In vivo inhibition of SARS‐CoV‐2 by PSS. A) In a prophylactic application scenario, K18‐hACE2 mice (6 male mice per group) received BS3 or S3 (5 µg i.n., half per nostril, 50 µL volume in total) 60 and 10 min prior to infection. For infection, SARS‐CoV‐2 Wuhan Hu‐1 was inoculated i.n. (300 FFU). All animals were sacrificed 2 dpi and lungs were harvested. After tissue homogenization, viral RNA levels B) and nucleoprotein levels C,D) were analyzed by RT‐qPCR and western blot, respectively. E) In a pre‐incubation scenario, SARS‐CoV‐2 Wuhan Hu‐1 (300 FFU) was mixed with polymers (100 µg mL^−1^ concentration on virus) and the mixture was applied i.n. (50 µL total volume). At seven hpi, mice were further treated i.n. with 5 µg polymer in 50 µL volume. Mice were sacrificed and lung tissue was analyzed for viral RNA and protein two dpi (F–H). Gray values of WB images for tubulin (cellular control) and SARS‐CoV‐2‐N bands were quantified by Fiji. Error bars show means ± SEM of six animals (*n* = 6). Ordinary one‐way ANOVA with Dunnett's post‐test compares mean viral loads and viral protein levels of treated animal groups to PBS‐treated control animals. ** = *p* < 0.01. Schematics in (A) and (E) created with BioRender.com

The in vivo activity of the compounds against SARS‐CoV‐2 was first evaluated in a prophylactic scenario (**Figure** **12**): hACE2 transgenic mice received BS3, S3, or vehicle (PBS) 60 and 10 min before infection, at 5 µg mL^−1^ (25 µL) total volume. At the same volume, mice were then intranasally infected with 300 FFU SARS‐CoV‐2. Body weight and clinical symptoms were monitored daily until the animals were sacrificed two days after infection and homogenized lung tissues were subjected to RT‐qPCR to determine viral RNA levels and western blot to determine viral protein levels. No antiviral effect of BS3 or S3 (polymer) was observed in this scenario as no differences in viral RNA or protein levels (Figure 12B–D) could be observed between groups.

To determine if PSS exerts antiviral activity in vivo in a different treatment protocol, virions were incubated with the polymer or the core–shell formulation at a 0.1 g L^−1^ concentration for 1 min at 37 °C. Thereafter the mixture was administered intranasally at a dose of 5 µg per mouse. Additional polymer dose was administered after 7 h, and 48 h postinfection mice were sacrificed (Figure 12E). Quantification of the viral genome copies indicated a 240‐fold decrease in the viral load in case of the core–shell particle inhibitor, and a 550‐fold decrease in the case of PSS taken as a soluble polymer (Figure 12F). These data were confirmed by the western blot quantification of the SARS‐CoV‐2 nucleoprotein, which indicated a significant loss of the viral protein to a level below observable (Figure 12G,H). Thus, PSS and AuNP‐coupled PS reduced infection in vivo if administered simultaneously, followed by an additional postexposure application.

## 3 Conclusion

Taken together, results presented in this study illustrate that inhibitors of virus cell entry based on PSS and its core–shell formulations with AuNP represent promising candidates of highly sought‐after broad‐spectrum antiviral agents. These agents are active against a broad range of enveloped viruses, including HIV‐1, ZIKV, HSV‐1, RSV, seasonal coronaviruses and SARS‐CoV‐2 variants responsible for the current pandemic. We also show that for the low molar mass polymers, formulation as core–shell nanostructures significantly enhances the antiviral effects. We established the maximum tolerated dose of PSS in mice and revealed that at this dose, intranasal administration of the macromolecular inhibitor as well as its core–shell nanoformulations decrease RSV and SARS‐CoV‐2 viral loads in the lungs of mice. We acknowledge that as dosed, macromolecular inhibitors synthesized in this work were ineffective in preventing SARS‐CoV‐2 infection of mice in a prophylactic setting. In part, this may be because we used transgenic mice, engineered to ubiquitously express human ACE2 and be extremely susceptible to viral infection.^[^ ^46^, ^47^ ^]^ Second, drop‐wise intranasal administration in mice likely leads to the inferior distribution of the polymer compared to typical nasal sprays used in clinics, possibly limiting antiviral effects. A substantial volume fraction is also likely aspirated into the lungs shortly after application,^[^ ^48^ ^]^ possibly leading to subinhibitory concentrations in the upper respiratory tract. Nevertheless, our data provide strong proof‐of‐concept evidence for in vivo efficacy of the designed polymers and nano‐formulations as antivirals against respiratory viral pathogens.

To our best knowledge, the polymers presented herein are the first broad‐spectrum antiviral agents that suppress RSV and SARS‐CoV‐2 replication upon intranasal delivery in vivo, suggesting their further development as antiviral nasal spray and/or inhalation device. This route of application might be associated with lower site effects as compared to other broad‐spectrum antivirals that are to be administered systemically.^[^ ^49^, ^50^, ^51^, ^52^ ^]^ Moreover, their unique broad‐spectrum activity against enveloped viral pathogens renders our macromolecular inhibitors candidates for pandemic preparedness to combat emerging viruses, against which no preventative or therapeutic measure exist. We are now investigating the antiviral effects of the polymers in larger animals using commercial nebulizers for drug and viral administration.

## 4 Experimental Section

All reagents were purchased from Sigma‐Aldrich and used without further purification, unless mentioned.

### NMR Spectroscopy

^1^H NMR was recorded on a Varian INOVA 400MHz NMR spectrometer at 25 °C.

### Size‐Exclusion Chromatography

SEC was performed using a system comprising a LC‐20AD Shimadzu HPLC pump, a Shimadzu RID‐10A refractive index detector, and a DAWN HELEOS 8 light scattering detector along with a SPD‐M20A PDA detector, equipped with a HEMA‐Bio Linear column with 10 µm particles, a length of 300 mm, and an internal diameter of 8 mm from MZ‐Analysentechnik in series with a OHpak SB‐803 HQ Shodex column with the dimensions 8.0 × 300 mm a particle size of 6 µm, providing an effective molecular weight range of 1000–1 000 000 Da. The solvent used was 0.01 m PBS filtered through a 0.1 µm filter with 300 ppm sodium azide. Refractive index increment values (d*η*/d*c*) used to calculate the molecular weights of the polymers were determined by assuming full mass recovery.

### Synthesis of Poly(styrene sulfonate)

Sample S3: Sodium 4‐vinylbenzenesulfonate (1057 mg, 5.15 mmol, 1200 equiv.), 2‐(dodecylthiocarbonothioylthio)‐2‐methylpropionic acid (DCMA) (1.56 mg, 0.00429 mmol, 1 equiv.), and 2,2′‐azobis(4‐methoxy‐2.4‐ dimethyl valeronitrile) (V‐70) (0.19 mg, 0.00060 mmol, 0.14 equiv.) were dissolved in a DMF (1.7 mL) and water (1.3 mL) mixture. The mixture was transferred to an ampule and four cycles of freeze‐pump‐thaw were performed before the ampule was flame‐sealed. The polymerization was performed at 30 °C for 62 h. Upon completion, the ampule was opened and the crude mixture was analyzed with crude ^1^H NMR. The crude mixture was diluted with a NaHCO_3_/Na_2_CO_3_ buffer at pH 10.8 (15 mL) and purified by dialysis for 3 d followed by lyophilization yielding that wanted polymer (279 mg). Monomer conversion (NMR) 59%; ^1^H‐NMR (400 MHz, D_2_O) *δ* 7.54 (bs, 2H), 6.66 (bs, 2H), 1.39 (bs, 3H); Mn (SEC‐MALS) = 100 kDa and *Đ* = 1.1.

Samples with lower molar mass were synthesized via an identical protocol with the sole a variation being the monomer ratio to RAFT reagent. Sample S2: Mn (SEC‐MALS) = 38 kDa and *Đ* = 1.1. Sample S1: Mn (NNR) = 3 kDa.

### Nanoparticles Functionalization and Characterization

PSS polymers were conjugated via their RAFT‐end chains to different types of commercial gold nanoparticles (AuNP): spherical nanoparticles of 5, 10, 20, and 40 nm of diameter. To achieve this, polymer samples were directly dissolved in 2–3 mL of AuNP aqueous suspensions at a 2 g L^−1^ polymer concentration, and mixed during 48 h at 37 °C. The samples were purified by ultrafiltration (using Amicon Ultra Millipore filters) until the flow through liquid was pure from polymer, as monitored via UV–vis spectroscopy. The amount of polymer conjugated to AuNP surface was calculated by the difference between the initial added polymer and the amount of the nonconjugated polymers in the supernatant. For analysis of colloidal stability, polymer‐functionalized AuNP were freeze‐dried and dispersed in water, 10 phosphate‐buffered saline, or Dulbecco′s modified Eagle′s medium (DMEM) with the addition of fetal calf serum (FCS) at 10% v/v, followed by UV–vis characterization. TEM imaging of the samples was carried out FEI Tecnai G2 Spirit microscope.

### Antiviral Activity Testing

For testing antiviral activity of substances, generally, in virus treatment mode, the following protocol was used. Cells were seeded one day prior to the experiment to 96‐well plates and the next day, compounds were serially titrated in PBS and mixed 1:1 with virus. After incubating at 37 °C for 30 min, mixtures were added to target cell at 10‐fold dilution. After 1–3 d, infection rates were determined. Values were background‐subtracted and normalized to cells infected with virus treated with PBS only. Alternatively to virus treatment, cell treatment was conducted as following (for mechanistic experiments only). Compounds were titrated in PBS and added to cells directly, followed by 1 h at 37 °C. Virus was then added and as above, infection rates were detected after 1–3 d.

### HIV‐1

Virus stocks of HIV‐1 transmitted/founder virus (HIV‐1 M subtype C ZM247Fv‐1) were generated by transient transfection of HEK293T cells using Transit LT‐1 (Mirus). The supernatant was harvested two dpt, centrifuged 2000 × *g* 5 min and stored at ‐80 °C. For antiviral testing, virus stocks were incubated with compounds as described above and mixtures added to TZM‐bl cells seeded one day prior to transduction (10 000 cells). Virus input was chosen to reach ca. 50 000 RLU s^−1^ for virus incubated with PBS only. After 2 d, *β*‐galactosidase activity in cellular lysates was quantified by using the Gal‐Screen *β*‐Galactosidase Reporter Gene Assay System for Mammalian Cells (Thermo Fisher Scientific) and the Orion II microplate luminometer (Berthold, Bad Wildbad, Germany).

### ZIKV

Zika virus was grown on VeroE6 cells (grown in DMEM + 2.5% FCS + 100 units mL^−1^ penicillin, 100 µg mL^−1^ streptomycin, 2 × 10^−3^ m L‐glutamine, 1 × 10^−3^ m sodium pyruvate, and non‐essential amino acids) and harvested upon showing CPE, as described.^[^ ^53^ ^]^ Supernatants were centrifuged at 2000 × *g* for 5 min and aliquots were stored at ‐80 °C. VeroE6 was used for antiviral testing (6000 seeded), compounds were mixed with virus and the mixtures were added to cells resulting in MOI 0.2. The infection rates were determined after two days. Supernatants were collected and the cells were washed once with PBS Subsequently, 4% PFA in PBS was added to the supernatants and incubated for 30 min at RT. PFA was then removed and ice‐cold MeOH was added and incubated for 5 min at RT to permeabilize cells. MeOH was then aspirated and PBS was added. Next, cells were incubated with mouse anti‐flavivirus antibodies 4G2 in PBS containing 10% (v/v) FCS and 0.3% (v/v) Tween20 for 1 h at 37 °C. After washing with PBS containing 0.3% (v/v) Tween20, the cells were incubated with a horseradish peroxidase (HRP)‐coupled anti‐mouse antibody (1:20 000) for 1 h at 37 °C. Next, cells were washed four times and tetramethylbenzidine (TMB) substrate was added. After 5 min of incubation at room temperature, reaction was stopped with 0.5 m sulfuric acid. Absorption was measured at 450 nm and baseline corrected at 650 nm using an ELISA microplate reader.

### RSV

A549 cells (purchased from ATCC) were cultured in DMEM high glucose (Hyclone) supplemented with 5% FBS, 1% PEST, 1% L‐glutamine, 1% HEPES, and 1% sodium pyruvate. The cells were seeded the day before infection at 2.5 × 10^4^ cells per well in 48 well plates. RSV A expressing recombinant GFP were incubated with polymers (0.16–100 mg L^−1^) at 37 °C for 30 min prior to infection using MOI 1 of the pretreated virus. At 24 h postinfection, the cells were detached using Trypsin‐EDTA, washed and dead cells were stained with a LIVE/DEAD Fixable near‐IR Dead Cell Stain Kit (Life Technologies). Acquisitions were done on a FACSVerse Flow cytometer (BD Biosciences) and all analyses were performed with FlowJo software (Tree Star).

### HSV‐1

HSV‐1 F strain eGFP was grown on Vero E6 cells (grown in DMEM + 2.5% FCS + 100 units mL^−1^ penicillin, 100 µg mL^−1^ streptomycin, 2 × 10^−3^ m L‐glutamine, 1× 10^−3^ m sodium pyruvate, and nonessential amino acids) and harvested upon showing CPE, supernatants centrifuged at 2000 × *g* 5 min and aliquots stored at ‐80 °C. For antiviral activity, virus:compound mixtures were added to ELVIS cells seeded one day prior (5000 cells), resulting an MOI of 0.05, and infection rates determined 1 dpi by Gal‐Screen *β*‐Galactosidase Reporter Gene Assay System for Mammalian Cells (Thermo Fisher Scientific) and the Orion II microplate luminometer (Berthold, Bad Wildbad, Germany).^[^ ^54^ ^]^

### SARS‐CoV‐2

Viral isolate BetaCoV/France/IDF0372/2020 was obtained through the European Virus Archive global. Virus was propagated by inoculation of Vero E6 or Caco‐2 cells in 3.5 mL serum‐free medium containing 1 µg mL^−1^ trypsin.^[^ ^55^ ^]^ Cells were incubated for 2 h at 37 °C, before adding 20 mL medium containing 15 × 10^−3^ m HEPES. Three days postinoculation, when a strong cytopathic effect (CPE) was visible supernatant was harvested by centrifugation for 5 min at 1000 × *g* to remove cellular debris, and then aliquoted and stored at ‐80 °C. Infectious virus titer was determined as plaque‐forming units or TCID_50_. To determine antiviral activity of compounds against SARS‐CoV‐2, 25 000 Caco‐2 target cells were seeded in 96 well one day prior. Titrated compounds were mixed 1:1 with SARS‐CoV‐2, incubated for 30 min at 37 °C and mixtures added to cells, resulting in MOI of 0.0007. Two days later, infection was quantified by detecting SARS‐CoV‐2 N protein. Cells were fixed by adding 180 µL 8% PFA and 30 min of room temperature (RT) incubation. Medium was then discarded and cells permeabilized for 5 min at room temperature by adding 100 µL of 0.1% Triton in PBS. Followed by washing of the cells with PBS and stained with 1:5000 diluted mouse anti‐SARS‐CoV‐2 N antibody (Sinobiological, 40143‐MM05) in antibody buffer (PBS containing 10% (v/v) FCS and 0.3% (v/v) Tween 20) at 37 °C. After 1 h incubation at 37 °C, the cells were washed two times with washing buffer (0.3% (v/v) Tween 20 in PBS) before a secondary anti‐mouse antibody conjugated with HRP (Thermo Fisher #A16066) was added (1:15,000) and incubated for 1 h at 37 °C. Following three times of washing, the TMB peroxidase substrate (Medac #52‐00‐04) was added. After 5 min light‐protected incubation at RT the reaction was stopped using 0.5 m H_2_SO_4_. The optical density (OD) was recorded at 450 nm and baseline corrected for 620 nm using the Asys Expert 96 UV microplate reader (Biochrom). Values were corrected for the background signal derived from uninfected cells and untreated controls were set to 100% infection.

### hCoV‐229E and ‐OC43

Human common cold coronaviruses 229E and OC43 were grown on Huh7 cells as previously described.^[^ ^56^ ^]^ Antiviral activity was determined on Huh7 seeded one day prior (25 000 cells seeded) to incubation with viruses. Virus‐compound mixtures were added to cells resulting in MOI 0.002 (for 229E) and 0.006 (for OC43). Cells were fixed and permeabilized as described for SARS‐CoV‐2 and similarly, viral N protein was detected by specific primary and secondary HRP‐conjugated antibodies. For detection, TMB peroxidase substrate (Medac #52‐00‐04) was added and after 5 min light‐protected incubation at room temperature the reaction was stopped using 0.5 m H_2_SO_4_. Optical density (OD) was recorded at 450 nm and baseline corrected for 620 nm in an ELISA microplate reader. Values were corrected for the background signal derived from uninfected cells and untreated controls were set to 100% infection.

### IAV

Influenza strain A/PR/8/34 (H1N1; PR8) was purchased from ATCC and propagated in MDCK cells. Therefore, MDCK cells were inoculated with PR8 (MOI 0.005), which was diluted in cDMEM (DMEM supplemented with 0.2% bovine serum albumin (BSA), 2 × 10^−3^ m L‐glutamine, 100 U mL^−1^ penicillin, 100 µg mL^−1^ streptomycin, 25 × 10^−3^ m HEPES buffer and 1 µg mL^−1^ tosylsulfonyl phenylalanyl chloromethyl ketone (TPCK)‐trypsin). After 1 h incubation, the inoculum was removed by two times washing with PBS and afterward cDMEM was added. After 2 d cell suspension was harvested, sonicated for 10 min and frozen at ‐80 °C. The next day cell suspension was thawed on ice and afterwards centrifuged at 4 °C and 300 × *g* for 15 min. Supernatants supplemented with 0.5% BSA were frozen at ‐80 °C.

To determine the titer of IAV stocks, MDCK cells were seeded in 12‐well plates one day prior to infection. Cell culture supernatants were removed, cells were washed with PBS and 100 µL of serially diluted IAV stocks from 1:10^4^ to 1:10^9^ were added with 250 µL cDMEM. Infected cells were incubated for 1 h at 37 °C with regular shaking steps in between before 2 mL of overlay medium (cDMEM, 0.01% DEAE Dextran, 0.1% NaHCO3, 0.6% Avicel RC 581) was added to the cells. Three days postinfection, supernatants were removed and cells were fixed with 4% PFA for 1 h at RT. Cells were washed with PBS and incubated in crystal violet staining solution (0.5% crystal violet, 30% ethanol) for 10 min at RT. Cells were washed with H_2_O and dried until plaques were counted. The virus titer was calculated as plaque‐forming units per mL (PFU mL^−1^).

For testing antiviral activity, 20 000 CaCo cells were seeded one day prior infection. Virus‐compound mixtures were added to cells resulting in MOI 0.0007. 48 hpi, infection rates were determined by MUNANA (2′‐(4‐methylumbelliferyl)‐*α*‐D‐*N*‐acetylneuraminic acid)) assay, quantifying neuraminidase activity in cellular lysates. For this, cells were washed with PBS, lysed in 1% Triton X‐100 and 1:2 diluted in MES buffer (32.5 × 10^−3^ m MES monohydrate, 4 × 10^−3^ m CaCl2 dihydrate). 20 µL sample was incubated with 30 µL of MUNANA substrate (100 × 10^−6^ m) and incubated for 4 h at 37 °C and 190 rpm. 150 µL stop solution (0.1 m glycine in 25% ethanol) was added to the reaction before neuraminidase activity was determined using a Synergy H1 imaging reader (360 nm excitation and 450 nm emission).

### VSV Pseudoparticles with SARS‐CoV‐2 Spike Proteins

VSV pseudoviruses bearing glycoproteins of SARS‐CoV‐2 VOCs were prepared as previously described.^[^ ^40^ ^]^ Briefly, HEK293T cells were transfected with plasmids encoding SARS‐CoV‐2 VOC spike by Transit LT‐1 (Mirus).^[^ ^38^, ^39^, ^40^, ^41^ ^]^ Rhabdoviral pseudotype particles were prepared as previously described. A replication‐deficient VSV vector in which the genetic information for VSV‐G was replaced by genes encoding two reporter proteins, enhanced green fluorescent protein and firefly luciferase (FLuc), VSVΔG‐FLuc29 (kindly provided by Gert Zimmer, Institute of Virology and Immunology, Mittelhäausern, Switzerland) was used for pseudotyping.^[^ ^57^ ^]^ One day after transfection of HEK293T cells to express the viral glycoprotein, they were inoculated with VSVDG‐FLuc and incubated for 1–2 h at 37 °C. Then the inoculum was removed, cells were washed with PBS and fresh medium was added. After 16–18 h, the supernatant was collected and centrifuged (2000 × *g*, 10 min, room temperature) to clear cellular debris. Cell culture medium containing anti‐VSV‐G antibody (I1‐hybridoma cells; ATCC no. CRL‐2700) was then added (10% v/v) to block residual VSV‐G‐containing particles. Samples were then aliquoted and stored at ‐80 °C.

For pseudovirus inhibition experiments, Vero E6 were seeded in 96‐well plates one day prior to incubation with pseudoviruses (6000 cells per well). Pseudovirus stocks were mixed with serially titrated compounds and mixtures added to cells. After an incubation period of 16–18 h, transduction efficiency was analyzed. For this, the supernatant was removed, and cells were lysed by incubation with Cell Culture Lysis Reagent (Promega) at room temperature. Lysates were then transferred into white 96‐well plates and luciferase activity was measured using a commercially available substrate (Luciferase Assay System, Promega) and a plate luminometer (Orion II Microplate Luminometer, Berthold). For analysis of raw values (RLU/s), background signal of an uninfected plate was subtracted and values normalized to pseudoviruses treated with PBS only. Results are given as serum dilution resulting in 50% virus neutralization (NT50) on cells, calculated by nonlinear regression ([Inhibitor] vs. normalized response variable slope) in GraphPad Prism Version 9.1.1.

### TCID_50_ Endpoint Titration

To determine the tissue culture infectious dose 50 (TCID_50_), SARS‐CoV‐2 stocks were serially diluted 10‐fold and used to inoculate Caco‐2 cells. To this end, 20 000 VeroE6 cells were seeded per well in 96 flat bottom well plates in 100 µL medium and incubated over night before 62 µL fresh medium was added. Next, 18 µL of fivefold titrated SARS‐CoV‐2 of each dilution was used for inoculation on the cells in triplicates. Cells were then incubated for 5 d and monitored for CPE. TCID_50_/mL was calculated according to Reed and Muench.^[^ ^58^ ^]^

### Cytotoxic Activity Evaluation

Cytotoxic effects of compounds to target cells used for antiviral testing was performed using CellTiter‐Glo Luminescent Cell Viability Assay (Promega) as described by the manufacturer. Briefly, the supernatant was removed and reagent 1:1 diluted with PBS added. After incubation for 10 min at RT protected from light, supernatants were transferred to 96 well white plates and luminescence was recorded for 0.1 s using a plate luminometer (Orion II Microplate Luminometer, Berthold). Values were normalized to untreated cells.

### Nanoparticle Tracking Analysis

Nanoparticle tracking analysis (NTA) to determine the concentration and the size distribution of particles in saliva and EV isolations was performed using a ZetaView TWIN (Particle Metrix). Samples were diluted in particle‐free PBS and videos of the light‐refracting particles were recorded with the following settings: 25 °C fixed temperature, 11 positions, 1 cycle, sensitivity 85, shutter 100, 15 fps, 2 s videos/position, 3–5 measurements. The number, size distribution, and Zeta potential were evaluated by ZetaView Analyze 08.05.05 SP2. Between the samples, the chamber was thoroughly flushed with particle‐free PBS. To measure the apparent size of VLPs after incubation with compounds (60 min 37 °C), MLVgagYFP VLPs were tracked in 488 F‐NTA mode and sizes were determined by five acquisitions. For zeta potential analysis, VLPs incubated with compounds were diluted in ddH_2_O and zeta potential was measured in constant mode, five acquisitions in 488 nm F‐NTA per sample.

### SDS PAGE and Immunoblotting

Murine lung homogenates as prepared for quantification of viral loads by qPCR were used for the preparation of lysates for WB analysis. For the preparation of protein lysates, 10% (v/v) lysis buffer (10% Triton X‐100, 10% SDS) and protein inhibitor cocktail was spiked into homogenates, followed by high‐speed vortexing for 30 s and incubation at 4 °C for 5 min. Samples were then centrifuged at 21 000 × *g* for 20 min and supernatants were transferred to new tubes as protein lysates. Protein concentrations were determined by BCA Assay (Pierce Rapid Gold BCA Protein Assay Kit, ThermoFisher) and adjusted to the sample with the lowest concentration to achieve equal loading. Loading dye (4X Protein Sample Loading Buffer, Licor; final concentration 1X) and reducing agent (TCEP, Sigma Aldrich, final concentration 50 × 10^−3^ m) were added and samples were heated to 70 °C for 10 min. 15 µg protein were loaded per lane on Novex BisTris 4–12% Gels and separated in MES buffer (200 V, 30 min). Proteins were transferred to PVDF, membranes were then blocked in Casein Blocker for 1h at RT. Membranes were incubated in primary AB (SARS‐CoV‐2‐N: 40143‐R001 SinoBio) overnight, washed 3× in PBS‐Tween 20 (0.05%) and incubated with secondary StarBright‐coupled ABs and primary labeled anti‐Tubulin hFAB Rhodamine‐coupled (Bio‐Rad) antibodies for 1 h RT. After 4× washing, membranes were imaged on a Bio‐Rad ChemiDoc MP Imaging System. Intensity of bands was quantified by Fiji (ImageJ) built‐in gel analysis tool.

### Liposome Dye Leakage

Liposome leakage assay was performed as previously described.^[^ ^59^ ^]^ Liposomes for dye‐leakage assay were prepared by thin‐film hydration & extrusion. DOPC (1,2‐dioleoyl‐sn‐glycero‐3‐phosphocholine), sphingomyelin (Egg SM) and cholesterol (ovine wool) dissolved in chloroform (Avanti Polar Lipids, Alabaster, USA) were mixed at 45/25/30 mol% ratio in a glass round‐bottom flask. The solvent was then evaporated by a stream of nitrogen. The lipid film was then hydrated by adding 50 × 10^−3^ m 5(6)‐carboxyfluorescein prepared in 50% PBS (resulting in a solution iso‐osmolar to PBS) and adjusted to pH 7.4 with NaOH, yielding a total lipid concentration of 5 × 10^−3^ m. The flasks were shaken at 60 °C, 180 rpm, for 1 h. Small unilamellar vesicles were then prepared by 25× extrusion through 0.2 × 10^−6^ m polycarbonate membranes (Nuclepore Track‐Etched Membrane, Whatman, Maidstone, USA) in a Mini Extruder (Avanti Polar Lipids) on a heating platform at 60 °C. Free dye was removed by 2× size‐exclusion filtration using PD midiTrap Sephadex G‐25 columns (GE Healthcare, Buckinghamshire, UK) and liposomes were then quantified by nanoparticle tracking analysis (NTA) using a ZetaView (ParticleMetrix, Inning, Germany). For assay in 96‐well format, liposome preparations were diluted in PBS and 2.25 × 10^9^ per well added to plates in 90 µL volume. Fluorescence intensity was read in a Synergy plate reader (Biotek, Winooski, USA). Baseline was established by measuring fluorescence for 5 min, 10 µL of compounds then added and plates incubated for 30 min more with measurements every 1 min. Maximum intensity (100% dye release) was then measured by adding Triton X‐100 to 1% final concentration and again measuring for 5 min.

### Red Blood Cell Lysis Assay

Membrane‐disrupting activity of compounds was additionally studied by red blood cell lysis assay. Blood was drawn in EDTA‐containing tubes (Sarstedt), centrifuged 10 min at 1000 × *g* and 4 °C, serum removed and 1 mL PBS added for resuspension (ca. fivefold concentration from blood volume). Erythrocytes were counted using a Luna II automated cell counter (Logos Bio), diluted in PBS and ca. 3 million cells per well added to 96‐well plates in 50 µL volume. An equal volume of compounds or Triton X‐100 (1% v/v final concentration) was added and plates were shaken for 30 min at 500 rpm and RT. Plates were then centrifuged and supernatants transferred to a new 96‐well plate. Absorbance was measured at 405 nm; values were background‐subtracted (PBS only added) and normalized to Triton‐treated erythrocytes.

### RSV In Vivo

The in vivo studies were carried out in accordance with the INRAE guidelines, which are compliant with the European animal welfare regulation. The protocols were approved by the Animal Care and Use Committee at “Centre de Recherche de Jouy‐en‐Josas” (COMETHEA) under relevant institutional authorization (“Ministère de l’éducation nationale, de l'enseignement supérieur et de la recherche”), authorization number 201803211701483v2 (APAFIS#14660). All experimental procedures were performed in a biosafety level 2 facility.

Female BALB/c mice were purchased from the Centre d'Elevage R. Janvier (Le Genest Saint‐Isle, France). Mice at 8 weeks of age (*n* = 5 per group) were anesthetized with of a mixture of ketamine and xylazine (1 and 0.2 mg per mouse, respectively) and treated IN (intranasal) with 60 µL of rHRSV‐Luc (10^5^ p.f.u.) preincubated for 30 min in the presence of 12.5 µg of polymers. Two days postinfection (pi), mice were anesthetized and treated a second time by i.n. administration of 50 µL of 12.5 µg polymers diluted in PBS in final volume of 50 µL PBS. Luminescence measurement was then performed at four d.p.i.

### In Vivo Luminescence Measurements

Mice were anesthetized at 4 d.p.i. and bioluminescence was measured 5 min following IN instillation 50 µL of d‐luciferin (30 mg mL^−1^, Perking Elmer). Living Image software (version 4.0, Caliper Life Sciences) was used to measure the luciferase activity. Bioluminescence signals were acquired with an exposure time of 1 min. Digital false‐color photon emission images of mice were generated and show the average radiance (p/s/cm^2^/sr). Photons were counted within three different regions of interest corresponding to the nose, the lungs and the whole airway area. Signals are expressed as total normalized flux (p/s). Data were analyzed using the GraphPad Prism software version 6. The signals obtained for treated mice were compared with the RSV control using Mann and Whitney test.

### SARS‐CoV‐2 Infection Experiment in Mice

Transgenic K18‐hACE2 mice were obtained from a commercial supplier (Jackson Laboratory, USA) and bred at Fraunhofer IZI. Male mice (8–14 weeks old) were randomly assigned into groups of 6 animals and kept under standard conditions in isolated ventilated cages. Animal experiments were carried out under BSL3 conditions and performed in accordance with the EU Directive 2010/63/EU for animal experiments and were approved by local authorities. For prophylactic treatment, polymers (BS3 and S3) were intranasally applied 1 h and 10 min prior to infection at a concentration of 5 µg/50 µL. Afterward, mice were infected i.n. with 300 FFU SARS‐CoV‐2 Wuhan Hu‐1. In another experiment, mice were treated i.n. with polymers (BS3 and S3) pre‐incubated with 300 FFU SARS‐CoV‐2 Wuhan Hu‐1 for 1 minute at 37 °C at 5 µg/50 µL, and received a second intranasal treatment at the same dosage 7 hpi. The control group was infected with 300 FFU SARS‐CoV‐2 mixed with PBS and all intranasal treatment were performed under isoflurane anesthesia. Mice in both experiments were monitored daily for body weight and clinical score and euthanized on day 2 after virus inoculation. Lung tissues were collected in gentle MACS M tubes (Miltenyi Biotec, Germany) filled with 2 mL PBS on ice. The tissue was homogenized using gentleMACS Octo Dissociator (Miltenyi Biotec, Germany) with RNA_1 program. Afterwards, tissue homogenates were centrifuged at 2000 × *g* for 5 min at 4 °C to separate cell debris and the supernatant was removed and stored at ‐80 °C until viral RNA isolation. Viral RNA was isolated from 140 µL of homogenate supernatants using QIAamp Viral RNA Mini Kit (Qiagen, Germany) according to manufacturer's instructions. RT‐qPCR was performed using TaqMan Fast Virus 1‐Step Master Mix (Thermo Fisher) and 5 µL of isolated RNA as a template according to the published protocol.^[^ ^60^ ^]^ Ten‐fold serial dilutions in the range of 5 × 10^2^ to 5 × 10^6^ copies/5 µL of synthetic SARS‐CoV‐2‐RNA (Twist Bioscience, USA) were used as a quantitative standard to obtain viral copy numbers.

### Statistical Analysis

All data were analyzed using the GraphPad Prism software version 9.31. For in vitro infection experiments, the raw values obtained for uninfected cells were subtracted and values then normalized to untreated infection controls (set at 100%). All measured values were included without outlier removal. Values shown are means ± SEM unless indicated otherwise. Statistically significant differences between control and treatment groups were tested with ordinary one‐way ANOVA and Dunnett's post‐test. *P*‐values are shown as * = *p* < 0.05, ** = *p* < 0.01, *** *p* = 0.001, ns = not significant. The number of animals or repeated experiments is stated in each figure legend.

### Toxicology Study

All in vivo experimental procedures were approved by the regional animal experimental ethical review board in Stockholm, Sweden (17114‐2020). Male and female BALB/c mice, 7 weeks of age, were purchased from Charles River (Germany). Mice were anesthetized with isoflurane and polymers were administered intranasally in a volume of 25 µL (12.5 µL per nostril). Administration was performed once daily, for a period of 4 d. Animals were euthanized by cervical dislocation 24 h after the final administration and the following organs were excised and post‐fixed in 4% buffered formaldehyde: lungs, trachea, skull including nasal cavity and brain, esophagus, and stomach. Body weights and health status were recorded at administration and termination.

### Histopathology

Trachea, larynx, lungs, nasopharynx (3 levels, including nasopharyngeal duct and nasal associated lymphoid tissue) stomach, esophagus, and olfactory bulbs were subjected to histopathological analysis. Tissues were processed and embedded in paraffin, after which they were section (4–6 µm) and stained with hematoxylin and eosin. Tissue sections were examined by light microscopy. Histological changes were described according to distribution, severity (minimal, slight, moderate, marked and severe) and morphologic character.

## Conflict of Interest

The authors declare no conflict of interest.

## Supporting information

Supporting Information

## Data Availability

The data that support the findings of this study are available from the corresponding author upon reasonable request.
