# Supplementary material for: Macromolecular Viral Entry Inhibitors as Broad‐Spectrum First‐Line Antivirals with Activity against SARS‐CoV‐2
Source: Adv Sci (Weinh). 2022 May 11;9(20):2201378. doi: 10.1002/advs.202201378 (PMC9284172; doi:10.1002/advs.202201378)
Supplement: Supplementary file 1 — Supporting Information [file ADVS-9-0-s001.pdf]

ADVANCED SCIENCE
Open Access

Supporting Information

for *Adv. Sci.*, DOI 10.1002/advs.202201378

Macromolecular Viral Entry Inhibitors as Broad-Spectrum First-Line Antivirals with Activity against SARS-CoV-2

Rüdiger Groß, Lívia Mesquita Dias Loiola, Leila Issmail, Nadja Uhlig, Valentina Eberlein, Carina Conzelmann, Lia-Raluca Olari, Lena Rauch, Jan Lawrenz, Tatjana Weil, Janis A. Müller, Mateus Borba Cardoso, Andrea Gilg, Olivia Larsson, Urban Höglund, Sandra Axberg Pålsson, Anna Selch Tvilum, Kaja Borup Løvschall, Maria M. Kristensen, Anna-Lena Spetz, Fortune Hontonnou, Marie Galloux, Thomas Grunwald, Alexander N. Zelikin and Jan Münch**

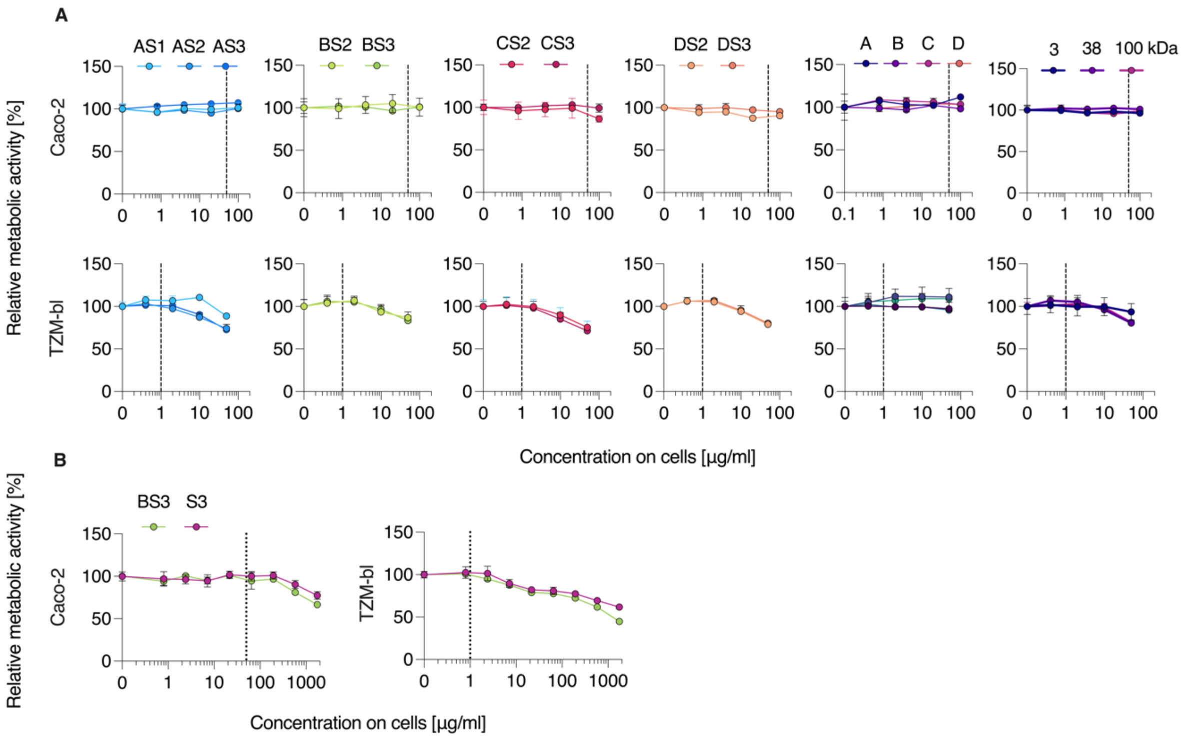

Figure S1: Effect of AuNP-conjugated and free PSS on cellular metabolic activity. (A) Compounds were dissolved and titrated in PBS before adding to Caco-2 (used for testing antiviral activity against SARS-CoV-2) or TZM-bl (used for testing antiviral activity against HIV-1) at indicated final concentrations on cells. Dashed vertical line indicates highest on-cell concentration of compounds during antiviral testing (considering 10-fold dilution of compound after pre-incubation with virus). Effects on cellular metabolic activity was determined by measuring intracellular ATP levels using CellTiter-Glo Luminescent Cell Viability Assay (Promega). One experiment (n = 1) in triplicates, means ± SD. **(B)** Dose-escalation and determination of effects on cellular metabolic activity for lead compounds BS3 and S3 on Caco-2 and TZM-bl cells; assay conducted as in (A). Triplicates, means ± SD.
